# 241 An All-Island Review of the Gaelic 4 Mothers & Others (G4M&O) Programme in Ireland

**DOI:** 10.1093/eurpub/ckae114.129

**Published:** 2024-09-26

**Authors:** Wesley O’Brien, Irene Hogan, Conor Philpott, Glynis Gardiner

**Affiliations:** University College Cork, Cork, Ireland; Munster Technological University Cork Campus, Cork, Ireland; University College Cork, Cork, Ireland; University College Cork, Cork, Ireland; Munster Technological University Cork Campus, Cork, Ireland

## Abstract

**Purpose:**

The Gaelic 4 Mothers & Others (G4M&O) initiative is a fun, non-competitive and recreational programme in Ireland for women to play Ladies Gaelic Football (LGF). The programme is aimed at women over 25 years of age who are not formally playing sport competitively. The initiative was introduced to community sports clubs in Ireland in 2008 by the Ladies’ Gaelic and Football Association (LGFA), and the programme now has over 500 sites across the island. The current study aims to assess the impact and benefits of the G4M&O initiative on; (i) participants, (ii) clubs and (iii) communities across Ireland. The research should inform the development of the G4M&O programme, providing critical insights for the LGFA and Sport Ireland.

**Project Description:**

As part of this all-island study, a 12-week pre-post longitudinal research design evaluation will be undertaken in 2024, using established qualitative and quantitative research methodologies focusing on: 1) Participants involvement within the club; 2) Participants involvement within the community. A nationally representative sample of participants (N = 300 targeted) will be recruited through the LGFA, which is the main national governing body (NGB) for LGF participation in Ireland.

A self-report validated online survey will be used to evaluate the impact of the programme on participants. Focus groups will also be conducted with a sample of participants (N = 40 targeted) across a variety of clubs exploring participants viewpoints and perspectives of the G4M&O programme. Data will be analysed quantitively using Statistical Package for the Social Sciences (SPSS) version 29 and qualitatively using Thematic Analysis (TA).

**Conclusions:**

This study will contribute to a qualitative and quantitative understanding of the benefits and impact of G4M&O programme in clubs and communities and should inform how the G4M&O initiative can be developed across Ireland over the next 10-years. The study will align to the Irish National Sports Policy 2018-2027, given that increasing physical activity and sports participation are the cornerstones of this new policy. The study may inform further research to develop a model for use in other sports.

